# Acceptability and Feasibility of a Smartphone-Based Real-Time Assessment of Suicide Among Black Men: Mixed Methods Pilot Study

**DOI:** 10.2196/48992

**Published:** 2024-01-22

**Authors:** Leslie B Adams, Thomasina Watts, Aubrey DeVinney, Emily E Haroz, Johannes Thrul, Jasmin Brooks Stephens, Mia N Campbell, Denis Antoine, Benjamin Lê Cook, Sean Joe, Roland J Thorpe Jr

**Affiliations:** 1 Department of Mental Health Johns Hopkins Bloomberg School of Public Health Baltimore, MD United States; 2 Centre for Alcohol Policy Research La Trobe University Melbourne Australia; 3 Department of Psychology University of Houston Houston, TX United States; 4 Department of Psychiatry and Behavioral Sciences Johns Hopkins School of Medicine Baltimore, MD United States; 5 Department of Psychiatry Cambridge Health Alliance Cambridge, MA United States; 6 George Warren Brown School of Social Work Washington University at St. Louis St. Louis, MO United States; 7 Department of Health, Behavior, and Society Johns Hopkins Bloomberg School of Public Health Baltimore, MD United States

**Keywords:** Black men, suicide, ecological momentary assessment, feasibility, acceptability, mixed methods, smartphone, real-time assessment, suicide prevention, user experience, behavior, implementation, intervention, mobile phone

## Abstract

**Background:**

Suicide rates in the United States have increased recently among Black men. To address this public health crisis, smartphone-based ecological momentary assessment (EMA) platforms are a promising way to collect dynamic, real-time data that can help improve suicide prevention efforts. Despite the promise of this methodology, little is known about its suitability in detecting experiences related to suicidal thoughts and behavior (STB) among Black men.

**Objective:**

This study aims to clarify the acceptability and feasibility of using smartphone-based EMA through a pilot study that assesses the user experience among Black men.

**Methods:**

We recruited Black men aged 18 years and older using the MyChart patient portal messaging (the patient-facing side of the Epic electronic medical record system) or outpatient provider referrals. Eligible participants self-identified as Black men with a previous history of STB and ownership of an Android or iOS smartphone. Eligible participants completed a 7-day smartphone-based EMA study. They received a prompt 4 times per day to complete a brief survey detailing their STB, as well as proximal risk factors, such as depression, social isolation, and feeling like a burden to others. At the conclusion of each day, participants also received a daily diary survey detailing their sleep quality and their daily experiences of everyday discrimination. Participants completed a semistructured exit interview of 60-90 minutes at the study’s conclusion.

**Results:**

In total, 10 participants completed 166 EMA surveys and 39 daily diary entries. A total of 4 of the 10 participants completed 75% (21/28) or more of the EMA surveys, while 9 (90%) out of 10 completed 25% (7/28) or more. The average completion rate of all surveys was 58% (20.3/35), with a minimum of 17% (6/35) and maximum of 100% (35/35). A total of 4 (40%) out of 10 participants completed daily diary entries for the full pilot study. No safety-related incidents were reported. On average, participants took 2.08 minutes to complete EMA prompts and 2.72 minutes for daily diary surveys. Our qualitative results generally affirm the acceptability and feasibility of the study procedures, but the participants noted difficulties with the technology and the redundancy of the survey questions. Emerging themes also addressed issues such as reduced EMA survey compliance and diminished mood related to deficit-framed questions related to suicide.

**Conclusions:**

Findings from this study will be used to clarify the suitability of EMA for Black men. Overall, our EMA pilot study demonstrated mixed feasibility and acceptability when delivered through smartphone-based apps to Black men. Specific recommendations are provided for managing safety within these study designs and for refinements in future intervention and implementation science research.

**International Registered Report Identifier (IRRID):**

RR2-10.2196/31241

## Introduction

Suicide among Black Americans is a critical public health priority that requires immediate attention. Studies have shown that suicide rates among this population are increasing and that Black men, in particular, are at a higher risk of suicide-related mortality [1,2]. It is crucial for public health efforts to prioritize suicide prevention in the Black community and address the systemic issues that contribute to mental health disparities [1,2]. Epidemiological data reveal that Black men die by suicide at rates 4 to 6 times greater than Black women and that suicide is the third leading cause of death for young Black men [2,3]. While nationwide suicide rates provide a broader perspective on this alarming public health issue, delving into state-specific data, such as in Maryland between 2016 and 2020, reveal a localized increase in suicide death for Black Americans, compared to their White counterparts and highlights the urgent need for tailored research priorities [4]. The COVID-19 pandemic further exacerbated these alarming trends, with Black Marylanders experiencing an uptick in suicide deaths during the initial lockdown periods in the United States [5]. These national and state-specific trends highlight the pressing need to address more robust approaches to suicide prevention, emphasizing the importance of ensuring equitable access to support and resources for all communities.

Mental health disorders, such as depression and substance use, are known risk factors for suicide among Black Americans [6,7], but recent research also highlights racism and associated contextual stressors as important, but underresearched, explanatory risk factors for suicidal thoughts and behaviors (STB) among Black adults [8-10]. Previous studies have demonstrated the significant impact of racism and associated daily stressors on mental health outcomes, including depression, anxiety, sleep disturbances, and posttraumatic stress disorder (PTSD), among Black individuals [11-15]. However, the emerging research highlights the need to examine the specific ways in which racism contributes to STB among this population. Further research in this area is crucial to identify effective prevention strategies that can reduce the impact of racism and mitigate the rising trend of suicide completion among Black adults.

Black Americans also face significant barriers in accessing mental health care due to stigma, systemic lack of access to services, and cultural mistrust [16-19]. Although Black men are approximately 30% more likely to report having a mental health condition compared to non-Hispanic White individuals [20], they also have significantly lower percentages of mental health-related visits prior to a suicide attempt than men of other racial and ethnic backgrounds and Black women [21,22]. In addition, traditional clinical records used to assess suicide risk and prevention often rely on distal and static measures of risk, such as prior diagnoses and family history, which may overlook the dynamic interplay of proximal factors that contribute to STB and patient-provider synergy. Consequentially, while Black Americans face considerable obstacles in accessing mental health care, these disparities are particularly pronounced among Black men, highlighting the urgency of addressing these disparities and adopting approaches to assess more proximal aspects of suicide risk and prevention.

Smartphone-based ecological momentary assessment (EMA) platforms are one such approach to collect dynamic, real-time data and encompass a range of different active and passive information, including, but not limited to, spatial trajectories (via GPS), physical mobility patterns (via accelerometer), social networks and social dynamics (via call and text logs and Bluetooth), and EMA surveys [23-25]. Understanding dynamic risk in suicide prevention efforts is important since decades of previous research have shown that single, static risk factors often add little to our understanding of who is at elevated risk and when [26]. More real-time data on STB may also provide key information about when to intervene with potential just-in-time interventions.

Although participant burden, noncompliance, and reactivity to the protocol measures have been cited as potential limitations to EMA approaches, studies using this methodology to assess STB demonstrate a favorable median response rate of 70%, suggesting feasibility [25,27-30]. These studies highlight the potential of EMA as a tool for capturing the complexity and variability of STB, ultimately aiding efforts to improve mental health outcomes. Specifically, one systematic review of EMA studies found suicidality fluctuates considerably over short periods of time and that those with higher levels of overall suicidality also have more fluctuations [27]. This review also noted risk factors such as negative affect, hopelessness, burdensomeness, and sleep characteristics that impacted suicidality [27]. In a sample of psychiatric inpatients, a separate study discovered that the use of real-time data collections significantly enhanced the accuracy of predictions for suicide attempts post-discharge [31]. Despite the promise of this methodology for the study and prevention of suicide [25,29,32], there has been no study, to date, that uses this approach to assess experiences among Black men at critical periods for early intervention [26]. Black men face daily societal and cultural stressors that may contribute to their heightened risk of suicide, including but not limited to, systemic and interpersonal racism, economic disparities, and limited access to mental health resources [8,33,34]. To address this gap, additional research is needed to clarify the efficacy of EMA monitoring as a suicide prevention tool for this at-risk population.

## Methods

### Eligibility and Recruitment Procedures

We recruited adult Black or African American men (18 years of age or older) with a lifetime history of suicidal ideation or attempt residing in Maryland counties where mobile crisis support was available. To be eligible, participants had to (1) own a smartphone and (2) not be present with active psychosis or cognitive deficits. We used two recruitment approaches: (1) providing recruitment information to eligible, active patients via the Johns Hopkins Health MyChart, the web-based patient portal of the Epic electronic medical record system that allows for health communication between patients and health care providers and (2) clinician referral. Participants who met the eligibility criteria through either of these approaches were referred to the study coordinator and were required to complete a screening survey to confirm their interest and eligibility.

### Ethical Considerations

The study was approved by the institutional review boards of Johns Hopkins Bloomberg School of Public Health (IRB 00013672). All participants were provided detailed information about the study procedures and expectations, risks, and benefits as part of the informed consent process conducted by a research coordinator.

### Baseline Survey

**Figure 1:**
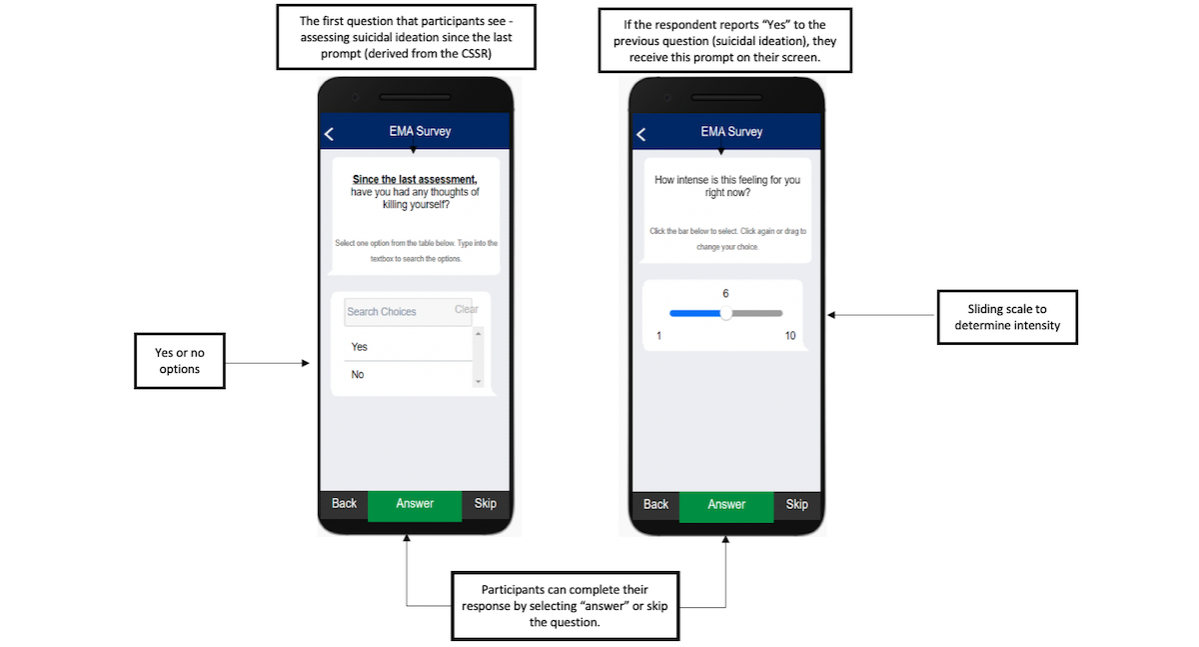
Screenshot of suicidal ideation and intensity questions on the ecological momentary assessment user interface. CSSR: Columbia Suicide Severity Rating Scale.

We asked enrolled participants to complete a brief baseline assessment including demographics and psychosocial measures associated with affective, gender, and race-specific factors associated with STB, such as anger, sadness, attributional style, and racial identity. A complete list of baseline measures is described elsewhere. During the informed consent process, participants received an overview of the MetricWire smartphone app from the study coordinator. The study coordinator also determined the county in which the participant resided, in order to align our safety protocol with mobile health crisis units in their area. After completion of the baseline survey, we asked participants to download the MetricWire app onto their personal smartphones for the duration of the study. The MetricWire app is available for both iOS and Android smartphone platforms at no cost in the Apple App Store (Apple Inc) or Google Play Store (Google), respectively. Examples of the user interface of the MetricWire app are presented in Figure 1.

### EMA and Daily Diary Data Collection Procedures

**Figure 2:**
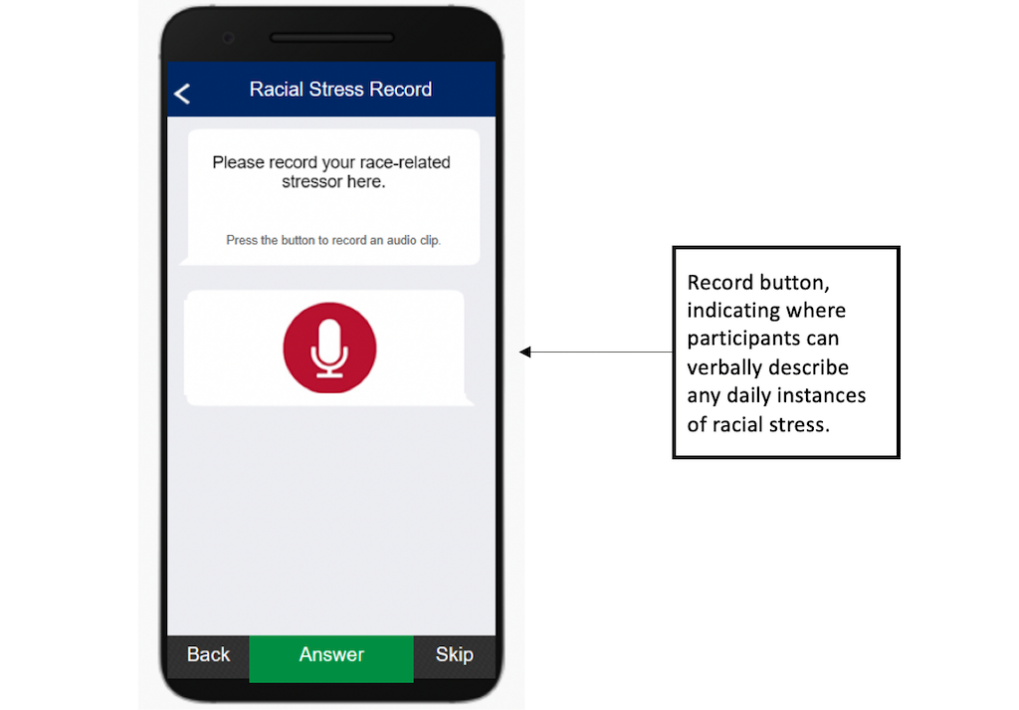
Screenshot of the respondent-driven racial stress recording option on the ecological momentary assessment user interface.

This study used the MetricWire app to deliver daily EMA surveys to participants at 4 time points between 10 AM and 6 PM. Each of the prompts was designed to occur at least 2 hours after the previous prompt and included 3 push notification reminders at 20, 40, and 55 minutes after the initial prompt. If the participant did not complete the EMA survey within 60 minutes, it was marked as incomplete. The EMA surveys were designed to be brief and take no more than 3 minutes to complete to reduce respondent burden. To capture instances of racism-related stressors occurring outside of the random EMA survey prompts, participants were allowed to record event-driven entries detailing their daily experiences (see Figure 2).

We administered a brief daily diary survey via the MetricWire app once per day to assess participants’ everyday experiences, including sleep-related impairment and quality, as well as their daily experiences with racism-related stress. The daily diary was prompted each day at 8 PM. This survey was not designed to capture momentary instances but rather to provide a more comprehensive picture of participants’ daily experiences.

### Exit Interview

After the 7-day data collection period, each participant underwent a qualitative semistructured exit interview conducted by the study team. The interviews probed participants on issues such as question difficulty and clarity, potential revisions to question prompts, and overall satisfaction with the study protocol and EMA surveys. Verbatim transcripts of the recorded interviews were produced and analyzed to evaluate the feasibility and effectiveness of the study protocol.

### Participant Incentives

To encourage higher EMA survey completion rates, participants were eligible to receive up to US $110 throughout the study duration. The incentive was incrementally phased, with participants receiving US $10 after completing the baseline survey, US $20 for completing 20% (7/35) of all surveys, US $50 for completing 50% (17.5/35) of all surveys, and US $80 for completing 80% (28/35) of all surveys. We offered an additional US $20 for the completion of the exit interview.

### Safety Protocols

Upon enrollment, we provided participants with a document containing information about local and national mental health resources, including suicide crisis hotlines, to support their well-being throughout the study. To ensure participant safety during the data collection period, we implemented a 3-tiered safety protocol. Moderate risk, which is defined as any suicidal ideations (“Have you had thoughts of killing yourself?”), since the last assessment, but without any plan or intent, resulted in a notification to the participant guiding them to the online support groups and community-based mental health services and urging them to seek support. Elevated risk, defined as suicidal ideation with intent or a plan within the last 24 hours (“Have you planned out how you would do it?” or “When you thought about making yourself not alive anymore, did you think that this was something you might actually do?”), resulted in the same notification that was given to participants with moderate risk and participants will be asked if they would like to handle the matter themselves or if they would like us to contact a mobile crisis response unit in their area on their behalf. Acute risk, defined as suicidal ideation with an action since the last assessment (“Did you do anything to make yourself not alive anymore or kill yourself?”), resulted in a call to the participant’s closest mobile crisis response unit made by a member of the study team on the participant’s behalf. To protect participant privacy, data were not stored on their smartphones. Instead, survey response data were automatically synced to the MetricWire servers when participants were connected to the internet, and encrypted response data were stored until the next connection. These servers were continuously backed up.

To provide additional support, all research personnel interacting with participants received training in psychological first aid to assist with identifying any mental health needs. EMA responses were closely monitored by the study coordinator and regular updates were provided during biweekly research team meetings between January 2022 and May 2023.

### Quantitative and Qualitative Data Analysis

To measure the acceptability and feasibility of the EMA method, we tracked four key metrics using Excel (Microsoft): (1) the proportion of eligible patients who enrolled in the study, (2) the percentage of completed EMA sessions out of the total number of scheduled sessions, (3) the number of safety-related incidents reported to mobile health crisis support teams, and (4) the average time taken to complete the EMA surveys.

Semistructured exit interviews were audio recorded and transcribed verbatim by the research team. The transcripts were then imported into Dedoose (version 8; Sociocultural Research Consultants, LLC) qualitative data analysis software for analysis. A deductive coding framework was used based on previous conceptual definitions related to implementation science. The research team independently reviewed the transcripts and generated a preliminary codebook based on a priori concepts. These concepts focused on prevailing definitions of acceptability, feasibility, adoption, and fidelity to the EMA methodology.

## Results

### Demographics

Participants ranged from 18 to 34 years of age, with an average age of 27 (SD 5.31) years. The majority (4/10, 40%) of our sample had completed a high school degree, followed by an associate or bachelor’s degree (n=2, 20%), while some completed high school (n=1, 10%), college (n=2, 20%), and a master’s degree (n=1, 10%). Participants lived across 3 counties in Maryland, including Baltimore, Anne Arundel, and Howard. Furthermore, participants identified as a spectrum of sexual orientations, including heterosexual (n=3, 30%), gay (n=2, 20%), bisexual (n=1, 10%), pansexual (n=2, 20%), and questioning (n=1, 10%). One participant chose not to disclose his sexual orientation.

### Feasibility of EMA and Daily Diary

The MyChart recruitment service identified and sent study information to 744 active patients residing in Maryland. A total of 58 individuals completed our interest survey. Of those, 53 (91%) were attributed to the MyChart recruitment service. Three submissions were from clinician referrals and 2 were from community outreach events. Out of the 58 individuals completing the interest survey, 10 (17%) participants were enrolled in the EMA study. The primary reasons for exclusion after completing the interest survey were active psychosis (n=19) and no history of suicidal thoughts or behavior (n=10). A total of 7 individuals were excluded because they reported having no mental health care provider.

The 10 enrolled participants completed 166 EMA surveys and 39 daily diary entries. A total of 4 (40%) out of 10 participants completed 75% (21/28) or more of the EMA surveys. The average completion rate of all surveys was 58% (20.3/35), with a minimum of 17% (6/35), and maximum of 100% (35/35). A total of 4 (40%) out of 10 participants completed at least 5 out of 7 daily diary entries. The response rate for each day of the week ranged from 64% (3.2 surveys submitted on average; day 1) to 54% (2.7 surveys submitted on average; days 6 and 7). All 10 participants completed qualitative exit interviews at the conclusion of the study. There were no safety-related incidents that required mobile health crisis service response teams. On average, participants took 2 minutes and 5 seconds to complete EMA surveys and 2 minutes and 43 seconds to complete daily diary surveys.

### Qualitative Findings From Exit Interviews

After coding, several key themes arose, including the study implementation, the iatrogenic effects of repeated suicidality assessments, and strategies to improve usability and effectiveness in suicide-related smartphone app development for Black men. We present key details and findings on these themes as follows.

### Study Implementation

Table 1 shows representative quotes that illustrate participant perspectives on adoption, acceptability, and feasibility to the study protocol.

**Table 1:** Qualitative codes and selected quotations related to implementation of smartphone-based ecological momentary assessment study.

Code	Key quotes	Key critiques or recommendations
Acceptability	“They're good questions. It makes sense. It's exactly the information that you'd be looking for.” [Participant 1] “I like how it did call my therapist, because I finally set up a meeting with her. But she, you know, was like, ‘Hey, um, you know, come in contact with me, because I know you're not doing well.’ And I was like, ‘Yeah, that's fair.’” [Participant 2] “I thought the questions were pretty straightforward.” [Participant 4] “I think for the most part, it was good, it was self-explanatory. It was easy to get to.” [Participant 5]	“The only thing that I can really think of as far as that is the hours...Maybe they're asleep, maybe they're working etc., etc.” [Participant 1] “I thought it shouldn't have been the same questions. It got boring and repetitive and seemed like more of a hassle than something that was going to help me, because it's annoying now, because it's the same questions.” [Participant 5]
Feasibility	“What were your opinions regarding you know, how many surveys you got per day?” [Moderator] “Um, that was fine. I was. I'm not sure if you remember, but I was a bit wary beforehand. I was worried that it might be a little bit too many, but no, it was fine. The amount was perfectly okay. The timing was fine too. Yeah, the timing was definitely fine as well.” [Participant 1] “The only thing that I can really think of as far as that is the hours but as I had mentioned, before we even started this, anyone who's downloading this resource is probably doing it with the intention on actually utilizing it. So I can't really see someone actively like disregarding the surveys, unless they either don't notice them or, well, yeah, unless they don't notice them or somehow it slips past their schedule. Maybe they're asleep, maybe they're working etc., etc.” [Participant 1]	“The wording was, yeah, as I was saying before, it was very, extremely clinical, which I mean, you're not really supposed to be beating around the bush as far as the stuff is concerned.” [Participant 1] Regarding racial stress record: “I really don't like the sound of my voice...like, ugh, don't like that, and eventually just stopped doing them. Because it's just like, it's easier to like, write things down. I guess.” [Participant 2] “Sometimes you miss an alert and yeah and that would be like, all day, that was my main focus, like oh my God, I got to remember my survey or whatever.” [Participant 5]
Fidelity	Prompts to complete surveys: “It was good, because some days I will remember, I have to take the survey so such and such time but other days, I wouldn’t to remember. I’m glad they, the prompts you know, were there.” [Participant 6]	“One of the things that was a little annoying was like oh, it would crash on me a lot. So I would have to re-open it, but when I would re-open it, it would take me right back to the question that it crashed on me on. So, I was like, ‘Oh, okay, that was convenient.’” [Participant 4] “it seemed like every time I tried to open the app, it wouldn't even open, like sometimes it...ugh like it was so frustrating, so I just wouldn’t try to do it for a while.” [Participant 6] Lack of use due to app issues: “I didn't always have like issues as far as that's concerned, I didn't really use it very often, explicitly, because around the time that I was doing the, well, around the time that I was, you know, taking part in this study, it was out of work.” [Participant 1]
Appropriateness	Timing of prompts: “I'm not sure if you remember, but I was a bit wary beforehand. I was worried that it might be a little bit too many, but no, it was fine. The amount was perfectly okay. The timing was fine too. Yeah, the timing was definitely fine as well.” [Participant 1] Completing racial stress record in a predominantly Black neighborhood: “I normally don't go through racial stuff, like I'm in a neighborhood is predominately Black. I see one White person a day, maybe.” [Participant 5]	N/A^a^
Adoption	“I found myself being very, at least the first or second time, I felt myself being very, how am I supposed to answer this? It's, it just felt strange that, well, yeah.” [Participant 1] “I think that the first couple of questions are pretty intense ones. So, what was interesting for me at first—so I was having like a very good week, so the questions were a little bit jarring almost, if you're like, in the middle of a good day, and then you get those questions.” [Participant 8]	Intention influenced by number of prompts: “It's not like, something like a text or the person will later call me and be like, ‘Hey, did you see my text?’ I'll probably completely forget it exists, which I think is what I did for like a lot of the surveys.” [Participant 2]

^a^N/A: not applicable.

Overall, participants found the frequency and timing of the EMA survey prompts to be appropriate. One participant commented that the questions provided “exactly the information needed,” while another appreciated the reminder to contact their therapist. Participants generally found the questions straightforward and easy to understand, and the survey itself was considered self-explanatory and easy to access. Participant 1 noted that they were initially concerned about the number of prompts, but ultimately found them to be manageable:

I'm not sure if you remember, but I was a bit wary beforehand. I was worried that it might be a little bit too many, but no, it was fine. The amount was perfectly okay. The timing was fine too.

However, some participants reported feeling that the racial stress record and daily diary questions regarding discrimination may not have been relevant to their daily experiences.

I normally don't go through racial stuff, like I'm in a neighborhood that is predominately Black. I see one White person a day, maybe.

Participant 5

Our study identified some challenges related to technology issues that impacted the fidelity of our study implementation and, ultimately, participants’ ability to complete the surveys in a timely manner. Participants identified app crashes as a significant issue, which led to frustration and difficulty in completing the surveys.

One of the things that was a little annoying was like oh, it would crash on me a lot. So I would have to re-open it, but when I would re-open it, it would take me right back to the question that it crashed on me on. So, I was like, “Oh, okay, that was convenient.”

Participant 4

This issue not only caused inconvenience but also resulted in participants potentially losing progress on their survey responses. Consequently, this problem may have discouraged some participants from continuing with the study.

### Influence of Mood on EMA Compliance

The narratives shared by the participants highlighted the possibility of diminished effect resulting from frequent evaluations of suicidality, which could potentially exacerbate negative mental health outcomes. Participants’ accounts of their study experience suggest that depressed mood prior to a notification to complete a survey may impact compliance with suicide-related assessments. Overall, as stated by 1 participant, “during moments where I already wasn't feeling too great, it exacerbated things a bit.” Further, another participant in the study described how their survey compliance can be influenced by their mood:

It’s sort of like a reflex for like, whenever something buzzes on my phone, and I'm like, not in a good mood, I immediately, like, swipe it

get rid of the notification

Participant 2 noted that repeated assessments of suicidality could increase their awareness of passive suicidal thoughts, stating, “Not really trigger me, but be like, oh, yeah, I am, like, passively suicidal 99% of the time.” Another participant noted in his interview that he did not complete the last day of the trial due to an anniversary of a loved one’s death:

It was an emotional day and I didn’t want to continue because my responses would have changed drastically. I didn’t want nobody to look at me differently...I didn’t want to put myself in an uncomfortable position.

This response further cements the idea that response rates may be influenced by low mood. Several participants provided feedback on the survey questions, suggesting that shorter surveys may be more effective in assessing suicidality. They noted that if they responded with a positive mood score, assessed by the Patient Health Questionnaire-2 item measure (PHQ-2), it likely indicated they were doing well and not feeling suicidal. Multiple participants suggested that gauging mood before asking directly about suicide may improve the effectiveness of the survey.

Those questions are pretty good. But I would probably, like I'd probably try and, like keep the survey shorter...So maybe like gauge the mood before asking like, “Hey, are you going to kill yourself?” Because if I'm at like, 3, then the answer is like, it's no, it's, it's gonna be no, you know, because I'm in like, a pretty good place.

Participant 2

Given our study’s focus on suicide risk assessment, a significant portion of the survey questions pertained to assessing proximal or momentary risk for self-harm. Unfortunately, these questions were predominantly framed from a negative or deficit-oriented perspective, such as “Have you thought about killing yourself since the last prompt?” Several participants pointed out that this focus on negative experiences was contributing to their mood states and recommended that the survey be balanced with more positive questions.

It seems like everything was about if I have a terrible day...there should have been some questions that show record of your good days, too.

Participant 5

### Recommendations to Improve Usability and Effectiveness

As part of the qualitative exit interview, participants were asked to provide feedback and suggestions for the future use of the app. In relation to the app’s accessibility, several participants suggested incorporating features that would promote ease of use. Specifically, participants mentioned that a dark mode would be beneficial, as the bright white background of the app was uncomfortable for some. In addition, participants suggested incorporating a text-to-speech feature that would read the survey questions aloud to make it easier for individuals with visual impairments or reading difficulties.

To encourage better participation and compliance, some participants suggested that a more flexible schedule would better align with their daily routines and lifestyles. Some participants pointed out that they are less inclined to fill out surveys when they are occupied with other social activities. As a result, participants suggested a schedule that can be customized according to their personal needs and circumstances.

I guess, maybe if I could like, sort of choose my own schedule...like put it at times where I know that I'm going to be like, on my phone, or like, in my bed...I'm probably going to be in my bed not doing anything else, like probably playing a game on my phone. So I'll tell myself, “Hey, you have no excuse, fill this out so that they know you haven't, like died.” But, if it's like, in the middle of me, like going to the mall with my friends, I'm most definitely not going to stop and be like, “Hold on everybody, I have to fill this out” I'm just gonna forget about it.

Participant 2

Participants provided various suggestions for enhancing the interactivity of the smartphone-based app, including incorporating a journaling component to allow them to document their thoughts in written form. Participant 2 described the potential benefit of such a feature, stating that it could provide a private space for them to express their emotions and thoughts more freely, without the fear of judgment or negative consequences.

I was thinking, maybe something where, you know, if you're not feeling that bad, you can just write down something...And knowing that it's going to medical professionals, I probably will be as kind of open as I am in my journal.

Additionally, other participants expressed interest in in-app features that would allow for personalized coping strategies, such as guided breathing exercises or links to mental health resources. These in-app features were seen as potential tools to promote engagement with the app and ultimately improve mental health outcomes. Finally, participants recommended that the survey include questions that allow for a more comprehensive and nuanced picture of their mood and well-being, and that these questions should be presented before questions pertaining to suicide risk to “ease into the heavier stuff,” as suggested by participant 1.

## Discussion

### Principal Findings

Our study aimed to assess the feasibility of using smartphone-based EMA to evaluate suicidality in real time among individuals in a high-risk sample of Black men. During the interviews, most participants generally reported that the frequency and timing of the prompts and reminders were suitable for the project and their daily lives. The results indicate that this approach shows promise for future research in this population. The results also suggested that while suicide-related smartphone apps have the potential to be effective tools for suicide prevention, they need to be developed and implemented with care. Even though the completion rate for EMA surveys was not optimal, we are encouraged by the high level of compliance with the daily diary entries. The study also did not pose any safety concerns, and the completion time for the surveys was found to be relatively short. Participants reported that the questions were generally relevant to their daily experiences and easy to understand. Additionally, the user feature that allowed for open-ended audio recordings was found to be useful in capturing daily experiences of racism, which is an important factor that could contribute to suicidality in this population. Finally, the qualitative findings from exit interviews suggest that the study procedures were perceived by participants to be acceptable and feasible.

Despite the promising results, the study revealed that the compliance rate was lower than in other studies not focused on Black men that leveraged EMA approaches to assess suicide risk [25,28]. One possible reason for this finding is that participants encountered technological issues and cited them as reasons for disengagement from the study. Participants noted redundancy in survey questions, which can lead to survey fatigue and reduced engagement. This redundancy might have contributed to the lower completion rates observed in the study. Moreover, the frequency of prompts may have contributed to the overall low compliance rate. The participants recommended fewer prompts and more flexibility in timing to enhance compliance.

Our findings also highlight the need for careful consideration of the potential harms of repeated assessments of suicidality, which focuses on a negative framing of mental well-being, and the need for the development of personalized interventions to mitigate any negative effects. Specifically, participant narratives from our qualitative exit interviews suggest that individuals experiencing negative moods may be less likely to engage with suicide-related assessments delivered via smartphone app notifications. Additionally, respondents felt that the EMA survey questionnaire could be more balanced by including both questions that assessed suicide risk and positive emotions as protective factors. One such measure, the Reasons for Living Inventory (RLI), was developed to identify factors that serve as deterrents against suicidal behavior. This inventory assesses positive emotions that support an individual’s decision to avoid death by suicide, should such thoughts emerge. Including measures, such as the RLI, that ground Black men to life is vital for addressing mental health challenges and fostering well-being for this demographic. By understanding how to balance the benefits of suicide-related assessments with their potentially harmful impact, scholars will be more equipped to identify protective factors and develop tailored interventions that are more likely to be used by Black men.

Our findings have important implications for the use of EMA among Black men. Smartphone-based methods, such as EMA and daily diaries, offer a unique opportunity to assess and evaluate suicide risk in real time. This approach represents a significant advancement in capturing momentary phenomena that could aid in suicide prevention, such as (1) characterizing dynamic mood changes over a short time span, (2) untangling stressful and racialized daily experiences, and (3) transmitting critical health information to health care providers to support care management. By using this approach, researchers can gain a more nuanced understanding of the experiences of high-risk populations and provide timely culturally relevant interventions to prevent suicide [23,24,32,35].

Moreover, researchers should be aware of certain populations that may be cautious to participate in research, such as Black men, particularly when it involves the explicit tracking and reporting of their daily experiences. This hesitancy can be rational, given the history of systemic exploitation of Black communities in biomedical research [36]. Therefore, it is important for researchers to acknowledge and address these historical injustices to build trust and facilitate participation among these populations. Only by taking these steps can researchers ensure that their work is ethical, respectful, and ultimately beneficial to the communities they seek to serve [19,37,38].

Smartphone-based suicide prevention is also increasingly becoming a viable approach for combatting rising suicide rates, especially among young adults. The prevalence of health-related internet searches via smartphones, as evidenced by the high percentage of people who are 18-29 and 30-49 years of age and who use their phones for medical information, suggests that smartphones can be effective tools for suicide prevention. Additionally, the fact that Black and Hispanic Americans use their phones for medical information more frequently than White non-Hispanic Americans suggests that smartphone-based interventions may be especially important in addressing suicide risk in communities that experience higher levels of stigma and barriers to accessing traditional health care resources [39]. By leveraging the widespread use of smartphones, suicide prevention efforts can reach a larger and more diverse population, potentially reducing the burden of suicide on individuals, families, and communities.

### Limitations

There are several potential limitations to our study that should be noted. First, our primary recruitment method was through MyChart messaging, which may have limited the pool of potential participants to those who are more likely to have additional physical comorbidities. The recruitment approach may have influenced the response rate, since participants may not have had the same motivation to respond to an interest survey, compared to a direct referral from a medical professional. Second, all participants were required to own a smartphone, which may have excluded individuals who did not have access to this technology. Additionally, we excluded Black men with active psychosis, which may have further limited the diversity of our sample. Given the potential for overdiagnosis of psychosis in underserved communities [40], it is possible that we also excluded potentially eligible individuals. Our study was also geographically restricted to Black men receiving health care in Baltimore, Maryland, and its surrounding counties. Future studies should consider additional venues and settings to recruit Black men who are not engaged in psychiatric care, including but not limited to social media, advocacy groups, and peer-led and community-based organizations. Finally, our study involved a relatively small number of participants, with 9 eligible participants completing the EMA surveys. This limited sample size may not fully represent the diversity and complexity of experiences among Black men and can affect the generalizability of findings to a larger population. Future studies should expand from our preliminary findings with a sufficient sample size to statistically investigate predictors of low compliance and user uptake.

### Conclusions

This study is the first to our knowledge to address critical gaps in suicide research by incorporating EMA to improve the care for Black men who are at risk of suicide. Using EMA may be an important tool to help stem challenges to the timely assessment of suicide among Black men, who comprise the largest percentage of deaths by suicide (81%) within the Black community [4]. Our findings highlight how acceptable and feasible this method is for this high-priority population, as well as potential approaches to improve its fit.

Our study provides critical insights into the use of smartphones to capture real-time data for assessing the mental and emotional health of individuals in high-risk clinical samples of Black men. Our results highlight the suitability of EMA using smartphone-based approaches for studying sensitive topics related to suicide in vulnerable populations. Furthermore, the findings shed light on the next steps for creating more equitable suicide prevention approaches, including identifying areas of missing data and the cultural acceptability of smartphone-based tools for health promotion. However, further research is necessary to expand these tools to assess structural racism and other racialized factors that influence Black men's daily lives.

## Abbreviations

EMA: Ecological momentary assessment
PHQ-2: Patient Health Questionnaire-2
PTSD: posttraumatic stress disorder
RLI: Reasons for Living Inventory
STB: suicidal thoughts and behavior

## Data Availability

The data set generated and analyzed during this study are available from the corresponding author on reasonable request.
